# Prevalence of the Use of Herbal Medicines among Patients with Cancer: A Systematic Review and Meta-Analysis

**DOI:** 10.1155/2021/9963038

**Published:** 2021-05-17

**Authors:** John Baptist Asiimwe, Prakash B. Nagendrappa, Esther C. Atukunda, Mauda M. Kamatenesi, Grace Nambozi, Casim U. Tolo, Patrick E. Ogwang, Ahmed M. Sarki

**Affiliations:** ^1^Department of Pharmacy, Faculty of Medicine, Mbarara University of Science and Technology, Mbarara, Uganda; ^3^The University of Transdisciplinary Health Sciences and Technology, Bangalore, India; ^2^Faculty of Nursing and Health Sciences, Bishop Stuart University, Mbarara, Uganda; ^4^School of Nursing and Midwifery, Aga Khan University, Uganda Campus, Kampala, Uganda; ^5^Family and Youth Health Initiative, Dutse, Jigawa, Nigeria

## Abstract

**Background:**

Although herbal medicines are used by patients with cancer in multiple oncology care settings, the magnitude of herbal medicine use in this context remains unclear. The purpose of this review was to establish the prevalence of herbal medicine use among patients with cancer, across various geographical settings and patient characteristics (age and gender categories).

**Methods:**

Electronic databases that were searched for data published, from January 2000 to January 2020, were Medline (PubMed), Google Scholar, Embase, and African Index Medicus. Eligible studies reporting prevalence estimates of herbal medicine use amongst cancer patients were pooled using random-effects meta-analyses. Studies were grouped by World Bank region and income groups. Subgroup and meta-regression analyses were performed to explore source of heterogeneity.

**Results:**

In total, 155 studies with data for 809,065 participants (53.95% female) met the inclusion criteria. Overall, the pooled prevalence of the use of herbal medicine among patients with cancer was 22% (95% confidence interval (CI): 18%–25%), with the highest prevalence estimates for Africa (40%, 95% CI: 23%–58%) and Asia (28%, 95% CI: 21%–35%). The pooled prevalence estimate was higher across low- and middle-income countries (32%, 95% CI: 23%–42%) and lower across high-income countries (17%, 95% CI: 14%–21%). Higher pooled prevalence estimates were found for adult patients with cancer (22%, 95% CI: 19%–26%) compared with children with cancer (18%, 95% CI: 11%–27%) and for female patients (27%, 95% CI: 19%–35%) compared with males (17%, 95% CI: 1%–47%).

**Conclusion:**

Herbal medicine is used by a large percentage of patients with cancer use. The findings of this review highlight the need for herbal medicine to be integrated in cancer care.

## 1. Introduction

Cancer is a major global health problem. In 2018, there were an estimated 9.6 million cancer fatalities and 18.1 million newly diagnosed cases [1]. Current trends indicate that the previously predicted increase to 20 million new cases by 2025 is likely to be surpassed [2]. The overall implications of this high rate of new cases of cancer include increased health, economic, and social costs, which will continue to put a burden on the limited resources and weak healthcare systems in poor countries. As a result, herbal (traditional) medicines use in cancer care may be increased in those countries.

Herbal medicine use associated with cancer, including in multiple oncology care settings, remains uncontested [3]. Previous studies have indicated that herbal medicine is the commonest form of complementary and alternative medicine (CAM) used by patients with cancer, with increasing use following a cancer diagnosis [4–12]. Furthermore, advances in conventional cancer care have not deterred patients with cancer from using herbal medicines for numerous reasons, including patient- or disease-related factors, cultural and historical factors, geographical or topological factors, and healthcare or system-related factors [11, 13–21].

Several studies have identified and documented the clinical (cancer disease) and individual (demographic) factors associated with the usage of herbal medicine in cancer. Numerous factors have been positively correlated with herbal medicine use in cancer, such as young age, high education level, high-income level, ethnicity, female gender, cancer diagnosis, longer survival period since cancer diagnosis, receiving single or multiple cancer chemotherapies, being married, completion of conventional cancer treatment, having certain cancer symptoms, cancer metastasis, and belonging to specific social groups [22–27]. However, old age, being a child, having cancer comorbidities, place of residence, and the experience of chemotherapeutic side effects are negatively associated with herbal medicine use in cancer [16, 26, 28].

Some herbal medicines possess compounds that are pharmacologically active against cancer cells, and preclinical studies have consistently shown that numerous herbal medicines or herbs have antiapoptotic, anti-inflammatory, cell regenerative, and antioxidant effects on cancer cells. However, the clinical evidence concerning the efficacy of most herbal medicines or specific herbs used in cancer is largely inconclusive [29, 30]. Clinical studies have reported that the use of herbal medicines in cancer lowered the mortality rate hazard ratio of patients with lung cancer (thereby increasing survival), improved patients' quality of life through reducing cancer symptoms and conventional drug side effects (e.g., nausea and vomiting), and had chemopreventive activity against certain cancers [17, 19, 31, 32].

Conversely, observational studies have indicated that concomitant herbal medicines use with antineoplastic drugs may result in drug to herb interaction (at all pharmacokinetic and pharmacodynamics levels) and adverse side effects or events [9, 31, 33, 34]. Adverse side effects or events range from minor side effects (e.g., gastrointestinal distress and allergy) to severe organ failure (e.g., hepatotoxicity, nephrotoxicity, bone marrow suppression, and respiratory and cardiac failure) [31, 32, 35–37]. Importantly, the release of antioxidants by herbal medicines is thought to reduce the oxidizing free radicals created by radiotherapy and chemotherapeutic agents against cancer cells, potentially reducing the effectiveness of conventional cancer treatment [24, 38]. Similarly, herbs commonly used in cancer such as St. John's wort and grape juice induce cytochrome isoenzymes (especially CYP3A4), which metabolize most conventional anticancer agents, thereby reducing the efficacy of targeted therapies such as tyrosine kinase inhibitors and anticancer hormonal therapies [31, 32, 38]. St. John's wort, specifically, was found to reduce the levels of plasma irinotecan, docetaxel, and imatinib mesylate antichemotherapeutic agents' concentrations [31]. Additionally, other herbs commonly used in cancer have been found to cause bleeding tendencies following surgery (e.g., ginkgo, garlic), hypoglycemia (e.g., ginseng), and hepatotoxicity (e.g., kava) and possess carcinogenic or negative tumor moderating effects [4, 35, 39]. In addition, heavy metal contamination in some herbal medicines may alter the pharmacokinetic profile of commonly used conventional cancer treatments [24, 38, 40].

Despite the widespread herbal medicines use among cancer patients, associated factors, and potential benefits and risks, the overall pooled prevalence of the use of herbal medicines among patients with cancer remains unclear. Previous systematic reviews focused on cancer used qualitative (narrative synthesis) designs and focused on synthesizing primary data on the use of CAM treatment modalities in general [41–47]. Therefore, we conducted a meta-analysis to explore the prevalence of the use of herbal medicine among patients with cancer across various geographical settings and patient characteristics and synthesized the literature on commonly used herbs in cancer. Understanding the prevalence of herbal medicine use by patients with cancer may help inform and guide healthcare policies geared toward integrating herbal medicines use in cancer care. Ultimately, this will help to improve outcomes for patients with cancer, develop wider public health (community) policies around herbal medicine legislation, and promote investment in education and research about herbal medicines used in cancer.

## 2. Methods

### 2.1. Protocol Registration

PROSPERO guidelines were used to develop this study protocol. The study protocol was subsequently registered with the open source foundation (doi: 10.31730/osf.io/cbtpy). The Preferred Reporting Items for Systematic Reviews and Meta-Analyses (PRISMA) guidelines are used to report this review' findings (https://www.prisma-statement.org, Supplementary Table 1).

### 2.2. Eligibility Criteria

We synthesized hospital- and population-based studies that reported the prevalence of the use of herbal medicines among patients with cancer (Table 1).

### 2.3. Information Sources and Search Strategy

We searched PubMed, Google Scholar, Embase, and African Index Medicus for articles published from January 2000 to January 2020. The key search terms words that were used to guide the search included the following: “Cancer” OR “Neoplasm” OR “Tumo*∗*” OR “Malignancy” AND “Herbs” OR “Herbal medicine” OR “Herbal material” OR “Herbal preparation” AND “Prevalence” OR “Use” OR “Proportion” OR “Percent*∗*” AND “Observational studies” OR “Cohort” OR “Cross-sectional*∗*” OR “Survey” OR “Cohort” (Supplementary Table 2). In addition, we manually skimmed the references of published review articles and primary studies to obtain any further eligible studies.

### 2.4. Data Extraction: Selection and Coding

After obtaining relevant studies (A.J.B), two authors (P.P.N and A.M.S) screened the identified articles' abstracts and titles and determined their eligibility for inclusion in this review, independently. Two authors (J.B.A and A.M.S) independently extracted the data using a standard data extraction form or tool created in Microsoft Excel 2016. Before data were extracted, the data extraction tool was pilot tested with 10 studies. Following the findings of the pilot test, improvements to the data extraction tool were made after reaching consensus with all reviewers. The extracted data included the following: (i) methodological or study characteristics, (ii) herbal medicine definitions or terms used, (iii) the focus of study (herbal medicine only or with other CAM modalities), (iii) use of conventional treatment, (iv) gender distribution, (v) participants' average age, (vi) sample size, (vii) proportion or frequency of herbal medicine use, (viii) herbs used in cancer, and (ix) conclusions. Countries were categorized by continent or world regions and according to World Bank economic indicators (Supplementary Table 3). The authors were able to resolve disagreements during data extraction through consensus.

### 2.5. Risk of Bias Assessment

Using the risk of bias of nonrandomized studies (RoBINS) tool, three authors (J.B.A, A.M.S, and P.P.N) independently assessed and reported the risk of bias in selected studies [48–50]. The risk of bias was categorized as high, moderate (unclear), and low across the various categories of bias (participation bias, selection bias, and confounder bias) (Supplementary Tables 4 and 5).

### 2.6. Data Analysis and Synthesis

The metaprop command in Stata software (version 12) was used to analyze the data. Freeman–Tukey double arcsine transformation was used to “stabilize the raw proportions” [51]. The pooled prevalence estimates and their confidence intervals (CI) were computed using the DerSimonian and Laird random-effects model (DL) and Wald method, respectively, based on “the transformed values and their variances” [51]. We inspected the forest plots for heterogeneity and then quantified this using chi-square tests and the *I*^2^ statistic. Because of significant heterogeneity (>50%), we explored the possible modifying effects of a number of study-level variables on the overall pooled prevalence of herbal medicine use among patients with cancer based on subgroup and meta-regression analyses [52, 53]. Modifying variables included the following: (i) year of publication (before or after 2010), (ii) study focus (herbal medicine alone vs. herbal medicine with other CAM), (iii) data collection method (researcher-administered vs. self-administered vs. document or record reviews), (iv) country income level (low and middle vs. high income), (v) study setting (hospital vs. community), (vi) study population (adults vs. children), (vii) cancer type (breast vs. prostate vs. hematological vs. others), (viii) region (continent), (ix) World Bank subregion, (x) study design (cross-sectional vs. cohort), and (xi) study country. We evaluated publication bias by inspecting the funnel plot for asymmetry and confirmed it using Egger's regression test [54]. We also reported the weighted pooled prevalence estimates and their 95% CIs. Data related to herbs used by patients with cancer were summarized and described.

## 3. Results

### 3.1. Study Selection Flow

**Figure 1:**
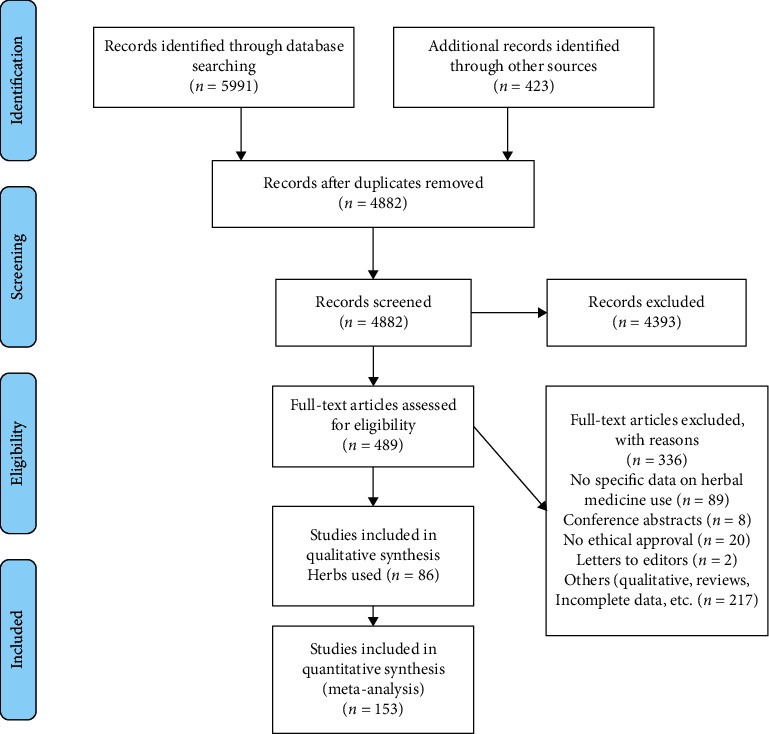
Study selection process based on PRISMA (https://www.prisma-statement.org).

In total, 6414 studies were retrieved from various search engines and databases (Figure 1). After eliminating duplicates, 4882 articles were selected for critical screening of the titles and abstracts. The final meta-analysis included 153 full-text articles that met the inclusion criteria. Eighty-six (86) articles were included in the qualitative synthesis of commonly used herbs by patients with cancer.

### 3.2. Study Characteristics

Characteristics of the included studies, which involved 809,065 participants (53.95% female) from 44 countries, are given in Table 2. The average age of the study participants was 50.98 ± 17.39 years, and the average response rate was 76.95% ± 19.78%. The majority of studies were conducted in America (34.19%), Europe (30.32%), and Asia (29.68%). However, based on World Bank subregions, most studies were carried out in Europe and Central Asia (32.92%), North America (30.32%), and East Asia and the Pacific (23.23%). The majority of the included primary research studies were from high-income countries (65.77%), with the US at the top of the list of individual countries (27.10%). Most studies used cross-sectional designs (99.35%), focused on studying herbal medicine as part of other CAM modalities (92.26%), and were conducted in hospital settings (85.81%) among adult populations (83.23%). Most participants were recruited using convenience sampling techniques (63.87%). The most common data collection method was self-administered interviews (52.90%), and the most common cancer type was breast cancer (18.71%).

### 3.3. Overall Pooled Prevalence of Herbal Medicine Use in Cancer by Income and Region

**Figure 2:**
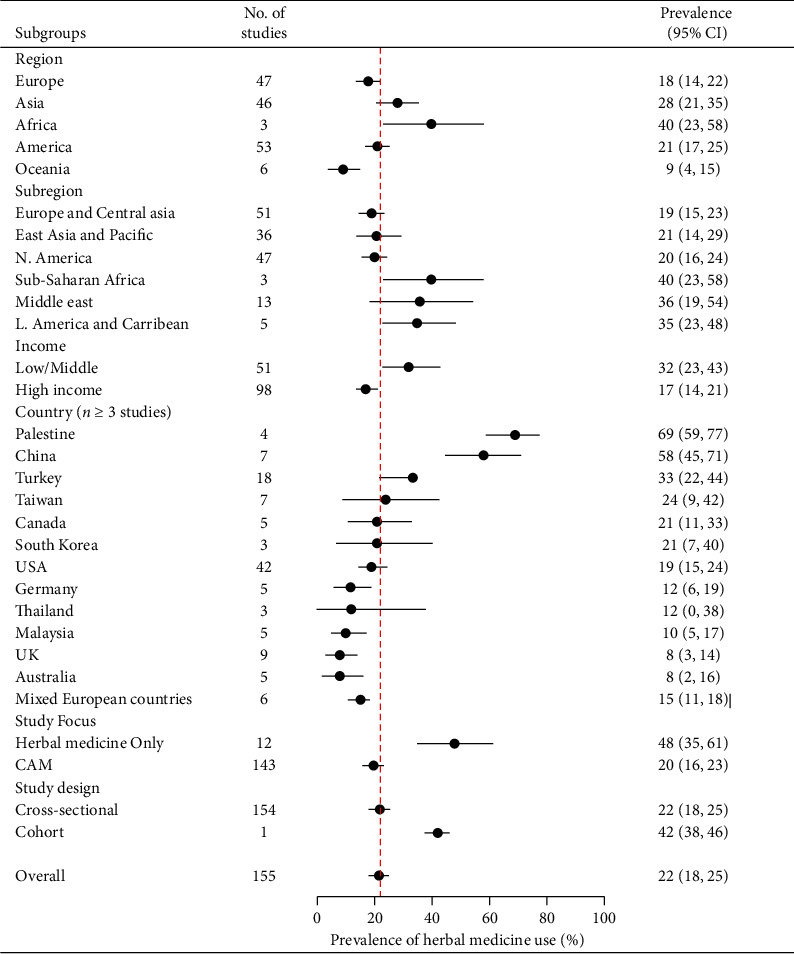
A summary (subgroup) forest plot on herbal medicine usage in cancer.

In total, 155 studies reported crude prevalence estimates of herbal medicine use by patients with cancer [3–40, 55–168]. The prevalence estimates ranged from 1% (95% CI: 0%-1%) to 93% (95% CI: 92%-93%). The overall random-effects pooled prevalence of herbal medicine use by patients with cancer was 22% (95% CI: 18%–25%, Figure 2). The *I*^*2*^ statistic was 99.84% (*χ*^*2*^ (df = 154) = 96436.14, *P* ≤ 0.001), indicating considerable heterogeneity among the included studies. On inspection, the funnel plot was symmetrical, as confirmed by Egger's regression test (*P*=0.063), indicating the absence of small-study effects (publication bias).

In terms of continents, the largest pooled prevalence of herbal medicine use among patients with cancer was found in Africa (40%, 95% CI: 23%–58%) and Asia (28%, 95% CI: 21%–35%, Figure 2). The lowest prevalence was recorded in Oceania (9%, 95% CI: 4%–15%). Analysis by subregion showed the highest prevalence of herbal medicine use among patients with cancer was in sub-Saharan Africa (40%, 95% CI: 23%–58%), followed by the Middle East (36%, 95% CI: 19%–54%), Latin America and the Caribbean (35%, 95% CI: 23%–48%), East Asia and the Pacific (21%, 95% CI: 14%–29%), North America (20%, 95% CI: 16%–24%), and Europe and Central Asia (19%, 95% CI: 15%–23%). Among selected countries (with *n* ≥ 3 studies), the highest pooled prevalence of herbal medicine use by patients with cancer was in Palestine (69%, 95% CI: 59%–77%, *n* = 4), followed by China (58%, 95% CI: 45%–71%, *n* = 7), Turkey (33%, 95% CI: 22%–44%, *n* = 18), Taiwan (24%, 95% CI: 9%–42%, *n* = 7), Canada (21%, 95% CI: 11%–33%, *n* = 5), South Korea (21% 95% CI: 7%–40%, *n* = 3), the US (19%, 95% CI: 15%–24%, *n* = 42), mixed European countries (15%, 95% CI: 11%–18%, *n* = 6), Germany (12%, 95% CI: 6%–19%, *n* = 5), Thailand (12%, 95% CI: 0%–38%, *n* = 3), Malaysia (10%, 95% CI: 5%–17%, *n* = 5), the UK (8%, 95% CI: 3%–14%, *n* = 9), and Australia (8%, 95% CI: 3%–16%, *n* = 5). Finally, the pooled prevalence of herbal medicine use in treating cancer was higher among patients from low- and middle-income countries (32%, 95% CI: 23%–42%) compared with high-income countries (17%, 95% CI: 14%–21%).

### 3.4. Overall Subgroup and Meta-Regression Analyses

We conducted subgroup and meta-regression analyses to investigate the influence of various patient and study characteristics on the overall observed prevalence estimates to explore the heterogeneity observed among the included studies. The subgroup analyses indicated that geographical region (*P* ≤ 0.001), subregion (*P* ≤ 0.001), income group (*P* ≤ 0.001), study focus or type (*P* ≤ 0.001), study country (*P* ≤ 0.001), and study design (*P* ≤ 0.001) had statistically significant moderating effects on the overall pooled prevalence of herbal medicine usage in cancer.

**Figure 3:**
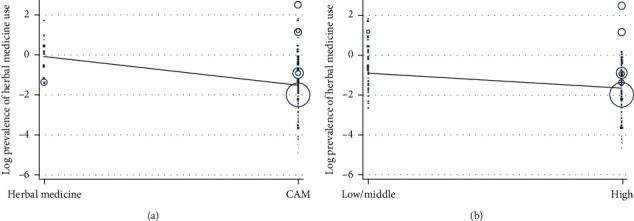
Meta-regression of herbal medicine use by study focus (a) and country income level (b), respectively.

Conversely, in the bivariate meta-regression analysis, only three variables were related to the overall pooled prevalence of usage of herbal medicine by cancer patients. Visual inspection of the scatter plot showed that studies that investigated herbal medicine in conjunction with other CAM modalities in cancer (20%, 95% CI: 16–23%) were more than twice less likely to report a higher pooled prevalence than studies that focused on herbal medicine use alone (48%, 95% CI: 35%–61%; *β* = −1.47, 95% CI: −2.18 to −0.76; *P* ≤ 0.001; Figure 3(a)). In addition, studies from high-income countries were less likely to report a high pooled prevalence of herbal medicine use in cancer compared to those from low- and middle-income countries (*β* = −0.803, 95% CI: −1.23 to −0.38; *P* ≤ 0.001; Figure 3(b)). However, there was a moderate positive relationship between subregion and pooled prevalence of herbal medicine use in cancer, with certain subregions being more likely than others to report a high prevalence of the use of herbal medicine in cancer (*β* = 0.134, 95% CI: 0.005–0.264; *P*=0.043).

### 3.5. Specific Pooled Prevalence by Study Subpopulation

#### 3.5.1. Pooled Prevalence of Herbal Medicine Use by Children with Cancer

**Figure 4:**
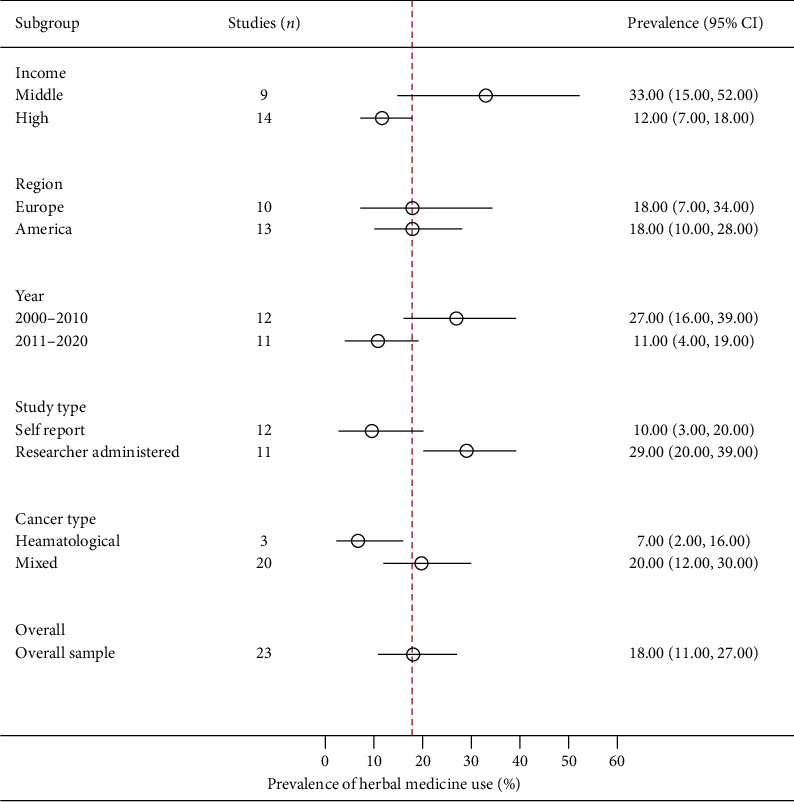
A summary (subgroup) forest plot on herbal medicine usage among children with cancer.

In total, the crude prevalence estimates of herbal medicine use by children with cancer was reported by 23 studies [30, 37, 40, 63, 82, 102, 106, 108, 118, 119, 121, 126, 132–135, 137, 143, 146, 151, 157, 165]. The prevalence estimates ranged from 1% (95% CI: 0%–5%) to 71% (95% CI: 61%–79%). With one exception, these studies were conducted in America and Europe. The overall random-effects pooled prevalence of herbal medicine use by children with cancer was 18% (95% CI: 11%–27%; Figure 4). The *I*^*2*^ statistic was 95.75% (*χ*^*2*^ (df = 22) = 517.11; *P* ≤ 0.001), suggesting considerable heterogeneity among the included studies. Egger's regression test confirmed that the funnel plot was asymmetrical (*P* ≤ 0.001), raising the possibility of small-study effects (publication bias).

Across income groups, low- and middle-income countries (33%, 95% CI: 16%–52%) were more likely to have a high prevalence of herbal medicine use by children with cancer than high-income countries (12%, 95% CI: 7%–18%). In terms of continents, Europe (18%, 95% CI: 7%–34%) and America (18%, 95% CI: 10%–28%) had a similar prevalence of herbal medicine use by children with cancer. Children with hematological cancers (7%, 95% CI: 2%–16%) were less likely to report the use of herbal medicine in cancer than those with all other cancer types combined (20%, 95% CI: 12%–30%).

Subgroup analyses indicated that the income group (*P*=0.02), data collection method (*P*=0.01), type of cancer (*P*=0.03), and study period (*P*=0.02) had significant moderating effects on the pooled prevalence of the use of herbal medicine in cancer. However, the meta-regression analysis showed that only three variables were statistically significant moderators of the pooled prevalence of herbal medicine usage in cancer. The income group was negatively related to the pooled prevalence of usage of herbal medicine in cancer, with studies from high-income countries less likely to report a high prevalence of the use of herbal medicine by children with cancer than those from low- and middle-income countries (*β* = −1.263 95% CI: −2.317395 to −0.208673; *P*=0.021). In addition, the use of herbal medicine was less likely to be reported in studies conducted between 2011 and 2020 (11%, 95% CI: 4%–19%) than those conducted between 2000 and 2010 (27%, 95% CI: 16%–39%; *β* = −1.19, 95% CI: −2.22 to −0.15; *P*=0.027). However, studies that used researcher-administered data collection instruments (29%, 95% CI: 20%–39%) tended to report a high pooled prevalence of herbal medicine use by children with cancer than those that were self-administered (10%, 95% CI: 3%–20%; *β* = 1.38967, 95% CI: 0.418–2.36; *P*=0.007).

#### 3.5.2. Pooled Prevalence of Herbal Medicine Use by Adult Patients with Cancer

Overall, 130 studies (that met the eligibility criteria) were incorporated in the meta-analysis of the prevalence of herbal medicine usage by adult patients with cancer [3–12, 14–29, 31–36, 38, 39, 55–59, 61, 62, 64–81, 83–101, 103–105, 107, 109–117, 120, 122–125, 127–131, 134, 136, 138–142, 144, 145, 147–150, 152–156, 158–164, 166–168]. The lowest crude prevalence was 1% (95% CI: 0%–1%) and the highest was 86% (95% CI: 78%–92%).

**Figure 5:**
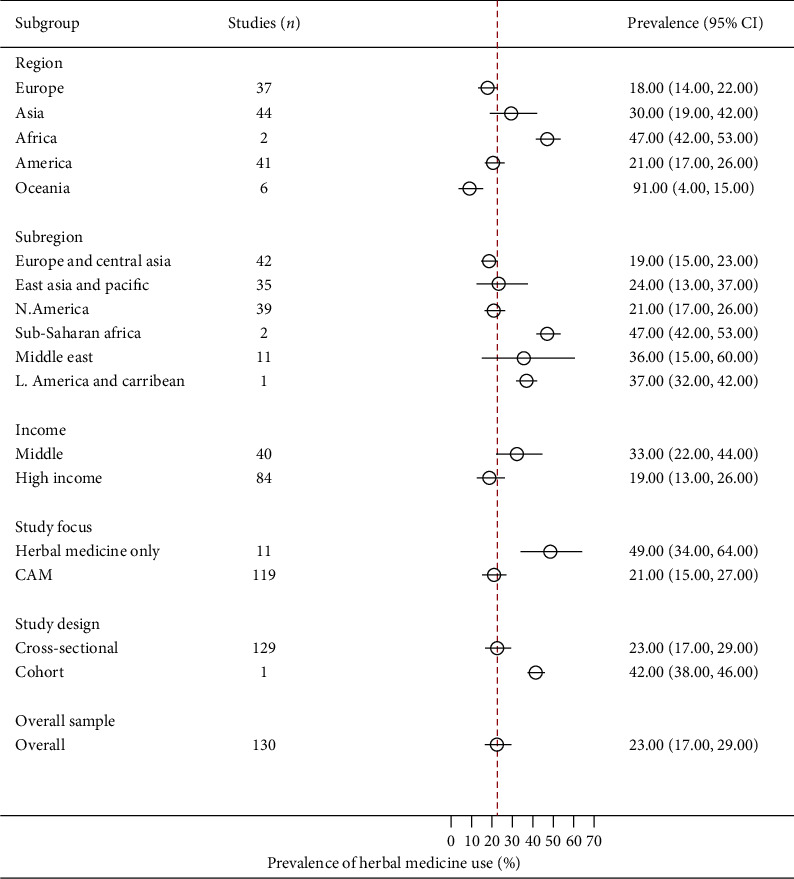
A summary (subgroup) forest plot on herbal medicine usage among adult patients with cancer.

The random-effects pooled prevalence of the usage of herbal medicine by adults with cancer was 23% (95% CI: 17%–29%; Figure 5). The *I*^*2*^ statistic was 99.96% (*χ*^*2*^ (df = 129) = 309703.50; *P* ≤ 0.001), demonstrating considerable heterogeneity among the included studies. The funnel plot was symmetrical as confirmed by Egger's regression test (*P*=0.220), suggesting there were no small-study effects (publication bias).

The highest pooled prevalence of the usage of herbal medicine in adults with cancer was in Africa (47%, 95% CI: 42%–53%), followed by Asia (30%, 95% CI: 19%–42%), America (21%, 95% CI: 17%–26%), Europe (18%, 95% CI: 14%–22%), and Oceania (9%, 95% CI: 4%–15%). In terms of subregions, the highest prevalence of herbal medicine use among adults with cancer was in sub-Saharan Africa (47%, 95% CI: 42%–53%), followed by the Middle East (36%, 95% CI: 15%–60%), East Asia and the Pacific (24%, 95% CI: 13%–37%), North America (21%, 95% CI: 17%–26%), and Europe and Central Asia (19%, 95% CI: 15%–23%). Across income groups, adults with cancer from low- and middle-income countries (33%, 95% CI: 22%–44%) were more likely to report a high prevalence of herbal medicine than those in high-income countries (19%, 95% CI: 13%–26%).

The subgroup analyses indicated that study country (*P* ≤ 0.001), study focus (*P* ≤ 0.001), study region (*P* ≤ 0.001), study design (*P* ≤ 0.001), income group (*P*=0.02), and study subregion (*P* ≤ 0.001) were statistically significant moderators of the pooled prevalence of herbal medicine usage by adults with cancer. However, bivariate meta-regression revealed that only study focus and income group had negative relationships with the pooled prevalence. Studies that focused on herbal medicine along with other CAM modalities (21%, 95% CI: 15%–27%; *β* = −1.506, 95% CI: −2.25 to −0.76; *P* ≤ 0.001) and those from high-income countries (*β* = −0.75, 95% CI: −1.23 to −0.27; *P*=0.002) were less likely to report a high pooled prevalence of herbal medicine use in adults with cancer than studies that focused on herbal medicine alone (49%, 95% CI: 34%–64%) and were from low- and middle-income countries.

### 3.6. Specific Pooled Prevalence by Gender

#### 3.6.1. Pooled Prevalence of Herbal Medicine Use by Male Patients with Cancer

**Figure 6:**
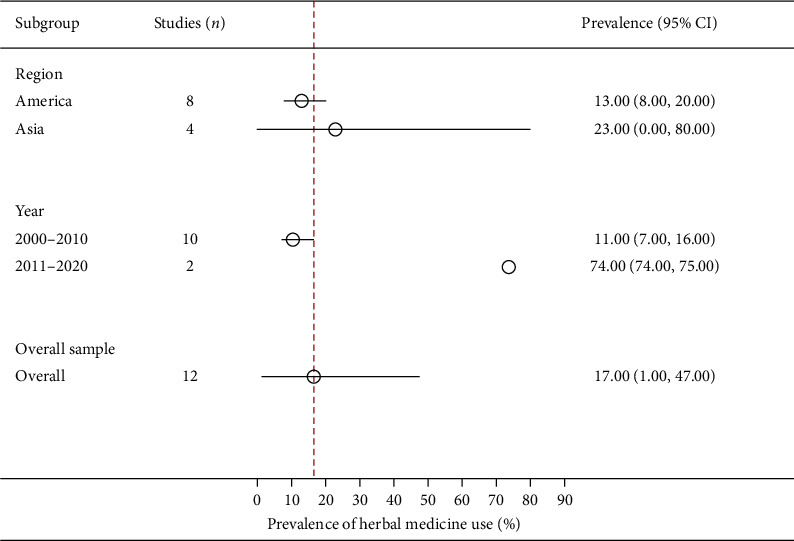
A summary (subgroup) forest plot on herbal medicine usage among male patients with cancer.

Twelve studies reported crude prevalence estimates of the use of herbal medicine among male patients with cancer [71, 76, 88, 90, 97, 101, 112–114, 152, 161, 167]. All of these studies were conducted among patients with prostate cancer in the Americas and Asia. The crude prevalence ranged from 1% (95% CI: 0%–7%) to 76% (95% CI: 75%–76%). The overall random-effects pooled prevalence of the use of herbal medicine by male patients with cancer was 17% (95% CI: 1%–47%; Figure 6). The *I*^*2*^ statistic was 99.98% (*χ*^*2*^ (df = 22) = 44251.66; *P* ≤ 0.001), representing significant heterogeneity among the included studies. The funnel plot was symmetrical as confirmed by Egger's regression test (*P*=0.064), indicating the absence of small-study effects (publication bias).

The continent of Asia (23%, 95% CI: 0%–80%) had a higher prevalence of the male patients with cancer who used herbal medicine compared with America (13%, 95% CI: 8%–20%). Subgroup analyses indicated that only the study period (*P* ≤ 0.001) had a statistically significant moderating effect on the overall pooled prevalence of the use of herbal medicine among male patients with cancer. Contrariwise, the meta-regression analysis showed that this relationship was weak, with studies conducted between 2011 and 2020 (74%, 95% CI: 74%–75%) more likely to report a high prevalence of the use of herbal medicine by male patients with cancer than those conducted between 2000 and 2010 (11%, 95% CI: 7%–16%; *β* = 2.06, 95% CI: −0.002 to 4.13; *P*=0.050).

#### 3.6.2. Pooled Prevalence of Herbal Medicine Use by Female Patients with Cancer

**Figure 7:**
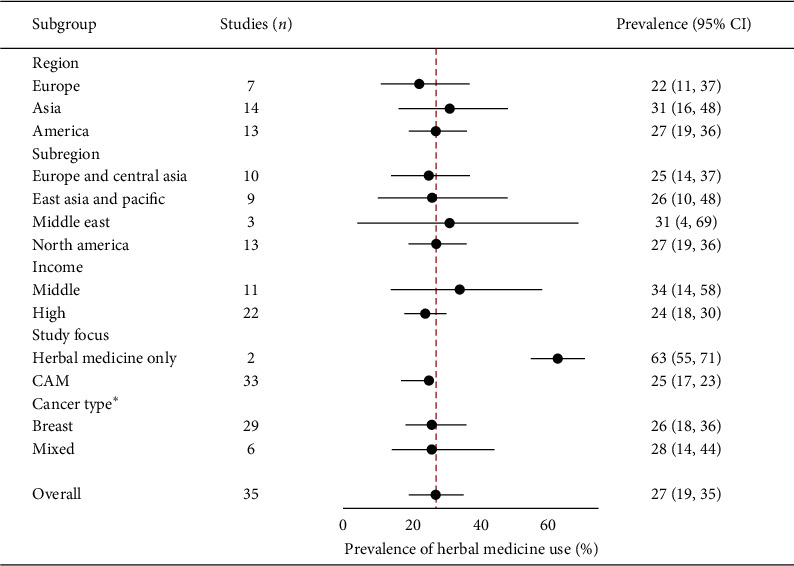
A summary (subgroup) forest plot on herbal medicine usage among female patients with cancer.

The prevalence of herbal medicine usage in female patients with cancer was reported in 35 studies [9, 10, 18, 24, 27, 28, 38, 55, 61, 64, 73, 83–85, 87, 90, 93, 95, 100, 110, 115, 123, 125, 138, 141, 145, 147, 149, 154, 155, 159, 163, 164, 166, 168]. With one exception, these studies were conducted in Asia, America, and Europe. The lowest prevalence of female patients with cancer using herbal medicine was 3% (95% CI: 2%–5%) and the highest was 85% (95% CI: 82%–87%). The random-effects pooled prevalence of the usage of herbal medicine by female patients with cancer was 27% (95% CI: 19%–35%; Figure 7). The *I*^*2*^ statistic was 99.63% (*χ*^*2*^ (df = 34) = 9133.34; *P* ≤ 0.001) reflecting considerable heterogeneity among the included studies. The funnel plot was symmetrical as confirmed by Egger's regression test (*P*=0.967*P*=0.967), indicating the absence of small-study effects (publication bias).

Among continents, the highest prevalence of the use of herbal medicine by female cancer patients was in Asia (31%, 95% CI: 16%–48%) followed by America (27%, 95% CI: 19%–36%) and Europe (22%, 95% CI: 11%–37%). The Middle East subregion (31%, 95% CI: 4%–69%) had the highest prevalence of the use of herbal medicine by female patients with cancer, followed by North America (27%, 95% CI: 19%–36%), East Asia and the Pacific (26%, 95% CI: 10%–48%), and Europe and Central Asia (25%, 95% CI: 14%–37%). A higher pooled prevalence of herbal medicine usage among female patients was recorded in low- and middle-income countries (34%, 95% CI: 14%–58%) compared with high-income countries (24%, 95% CI: 18%–30%). Similarly, studies involving patients with other cancers combined (28%, 95% CI: 14%–44%) tended to report a higher prevalence of herbal medicine usage among female patients with cancer than studies that focused on breast cancer alone (26%, 95% CI: 18%–36%).

The subgroup analysis showed that the study focus (*P* ≤ 0.001) and study region (*P* ≤ 0.001) had a statistically significant moderating effect on the overall pooled prevalence of the use of herbal medicine among female patients with cancer. However, in the meta-regression analysis, only study focus had a weak negative relationship with the pooled prevalence of herbal medicine use in cancer. Studies that focused on herbal medicine along with other CAM modalities (25%, 95% CI: 17%–33%; *β* = −1.634, 95% CI: −3.49–0.220; *P*=0.082; Figure 7) were less likely to have a high pooled prevalence than studies that focused on herbal medicine use in cancer alone (63%, 95% CI: 55%–71%).

### 3.7. Herbs Used and Reported by Patients with Cancer

In total, 86 studies reported herbs most commonly used in cancer, which included the following: evening primrose (*Oenothera biennis*), *Echinacea* (*Echinacea purpurea*), garlic (*Allium sativum*), stinging nettle (*Urtica dioica*), garden thyme (*Thymus vulgaris*), black cumin (*Nigella sativa*), green tea (*Camellia sinensis*), ginseng (*Panax ginseng*), ginger (*Zingiber officinale*), flaxseed (*Linum usitatissimum*), myrtle (*Myrtus communis*), ginkgo (*Ginkgo biloba*), aloe vera (*Aloe barbadensis*), St. John's wort (*Hypericum perforatum*), Essiac (containing four herbs: sorrel, slippery elm, Turkey rhubarb, and burdock), garden sage (*Salvia officinalis*), rosehip (*Rosa canina*), rosemary (*Rosmarinus officinalis*), turmeric (*Curcuma longa*), peppermint (*Mentha piperita*), Sabah snake grass (*Clinacanthus nutans*), kava kava (*Piper methysticum*), chamomile (*Matricaria chamomilla*), mistletoe (*Viscum album*), soy products (*Glycine max*), wild *Hedyotis diffusa*, barbed skullcap (*Scutellaria barbata*), noni (*Morinda citrifolia*), grape seed extract (*Vitis vinifera*), grapefruit (*Citrus paradisi*), milk thistle (*Silybum marianum*), French lavender (*Lavendula stoechas*), senna (*Cassia acutifolia*), licorice root (*Glycyrrhiza glabra*), cinnamon (*Cinnamonum zeylanicum*), snakehead (*Chana striata*), blackberry (*Rubus caesius*), saw palmetto (*Serenoa repens*), and wormwood (*Artemisia absinthium*). Other herbs that were less commonly used but reported by patients with cancer are listed in Supplementary Table 6.

## 4. Discussion

It has become increasingly common to base healthcare decision-making on information obtained from evidence-based medicine. Previously, this information was obtained from systematic reviews of interventional studies. However, this evidence is now also being acquired from systematic reviews of observational studies. The present review investigated the prevalence of the herbal medicine use among patients with cancer to inform and guide the development of healthcare policies concerning integrating herbal medicine in clinical cancer care.

This review suggested that a large estimated percentage of cancer patients use herbal medicine, especially during conventional treatment. The overall pooled prevalence of herbal medicine usage by patients with cancer was 22% (95% CI: 18%–25%), which means approximately one in five patients with cancer used herbal medicine(s) following a cancer diagnosis. This finding was consistent with the literature, where herbal medicine was reported as the leading form of CAM used in cancer [4–6, 8, 11, 12, 114]. This review also found that Africa and Asia had the highest pooled prevalence of the usage herbal medicine in cancer, with the lowest prevalence recorded in Oceania. Similarly, a larger percentage of patients with cancer from low- and middle-income countries used herbal medicine compared with those from high-income countries. This trend was repeated across specific subpopulations of children, adults, and female patients with cancer. The variation in prevalence across regions may be explained by variances in geographical characteristics (i.e., conditions that make some herbs easily available), cultural beliefs and attitudes, and liberalized or low regulation of herbal medicines [18–20, 31, 79, 131, 139, 162]. Conversely, the high herbal medicine usage in low- and middle-income countries might possibly be because of the low income levels, which may mean that patients with cancer are unable to pay for conventional cancer care (financial constraints) and or due to deeply rooted cultural practices related or favorable to use of herbal medicines. For example, as shown in this study, Asian countries such as South Korea and Taiwan, despite having the conditions and economic power to receive high-quality conventional therapies, patients from these countries still continue to use herbal medicine while accepting conventional therapies. Above all, high-income countries (of North America and Europe) where most studies included in this review were conducted do not possess a specialized or deeply ingrained traditional medicine use culture compared to countries of Asia and Africa.

The findings of this review suggested that compared to children (with cancer), adult patients with cancer were more likely to use herbal medicine, although this difference was not statistically significant. This variance in prevalence may be related to adults having more freedom to use herbal medicine than children who generally depend on their parents to access such products [106]. However, given the physiological nature of children (i.e., immaturity of organs such as the liver) and the potential risks of herbal medicine for children as reported in previous studies, parents need to make informed decisions based on evidence-based information before administering herbal medicine to their children to protect children from possible harmful effects [37].

We also found that more female patients with cancer compared to their male counterparts used herbal medicine. However, the pooled prevalence of the use of herbal medicine by male patients with cancer was from studies conducted among patients with prostate cancer; therefore, that reported prevalence best represents herbal medicine usage among patients with prostate cancer. Similarly, most studies that focused on female patients with cancer included patients with breast cancer, although the use of herbal medicine was higher in breast cancer than prostate cancer. These gender-based findings concur with previous literature, where the use of herbal medicine in cancer was related to being female, with women more likely to use herbal medicine as a primary mode of healthcare than men [22, 23, 138].

Finally, this review revealed several herbs commonly used in cancer, some with proven evidence of beneficial effects (anticancer effects) and others with potential risks (harmful side effects and drug interactions) to patients. Those with possible detrimental effects to patients included garlic, ginseng, kava, and St. John's wort [31, 32, 35]. Given that most of the studies that reported use of those herbs were conducted among cancer patients who were receiving conventional cancer therapies, clinicians (oncologists) should ask about herbal medicine use during their routine care of such patients.

### 4.1. Implications and Recommendations

Regardless of variation in the level of herbal medicine regulatory frameworks in different countries across the world, the high percentage of the usage of herbal medicine reported by this study calls for some form of integration of herbal medicine into cancer care. Healthcare providers must be at the center of this integration. The lack of sufficient clinical evidence should not be a deterrent to this integration, although health practitioners at all levels of patient care should routinely ask about, offer, and document evidence-based advice to patients with cancer on the safety and possible benefits of herbs and herb-drug interactions. Routine discussion of these issues during cancer screening, treatment, and follow-up may help to improve patient care outcomes. However, to equip health workers with evolving evidence on herbal medicines used in cancer, health educators need to continue incorporating knowledge about herbal medicines in oncological care training curricula and also develop programs geared toward understanding, evaluating, and validating herbal medicine use in cancer. In the short-term, health managers could develop short courses or refresher training for in-service healthcare workers on herbal medicines used in cancer to improve their knowledge on this subject.

In addition, policy makers at national governmental and international levels, such as drug authorities and health ministries, should incorporate and update new evidence regarding herbal medicine into oncology treatment guidelines, standard operating procedures, patient charts or electronic medical records, and pharmacopeias. This will assist healthcare workers to document herbal medicine practices in clinical care, which will subsequently promote clinical research on herbal medicine use in cancer.

As evidence regarding herbal medicine continues to evolve, policy makers in countries that regulate herbal medicine as dietary supplements or do not regulate herbal medicine at all need to update, review, or change their herbal medicine regulatory frameworks (either entirely or on a case-by-case basis) to protect patients with cancer from possible harmful effects posed by some herbs. In addition, as the media and other informal sources of information on herbal medicine are responsible for the high use of herbal medicine by cancer patients, oncology care centers and policy makers could create official websites or other media platforms with authentic and updated information on commonly or locally available herbal medicines to counter the misinformation from other sources. These platforms may be communicated to patients during routine cancer care. Importantly, these platforms should encourage patients to always seek advice regarding their specific circumstances from a qualified healthcare professional.

Successful integration of herbal medicine into cancer care either as an alternative form of medicine or alongside cancer medicine requires further high-quality multidisciplinary research on herbal medicines used in cancer, which requires research funding. Therefore, policy makers need to advocate, fundraise, and allocate resources for cancer research concerning herbal medicine use. For example, the lack of funding for research on herbal medicines may explain the relatively few published studies on herbal medicine in cancer, especially in Africa and South America, as observed in this study. In addition, it is important to note that in this study, we were unable to conduct a meta-analysis of the usage of herbal medicine in cancer across many other patients' characteristics due to inconsistent study variables. Therefore, it is necessary to develop a standardized survey tool customizable to various patients with cancer and settings to measure herbal medicine use in cancer. Such a tool will allow comprehensive systematic reviews to be conducted on this subject. It is also necessary to conduct more herbal medicine-specific observational research in cancer to obtain extensive statistics on the extent of herbal medicine usage in cancer across the world.

### 4.2. Limitations and Strengths of This Study

In terms of the quality of included studies, we rated the majority of studies as having a moderate to high risk of bias, which might have led to over- or underestimation of the pooled prevalence of herbal medicine use reported in this study. This was expected given that nearly all studies used cross-sectional designs. However, the majority of the included studies had moderately large sample sizes and high response rates. Second, there was a high proportion of heterogeneity (between-study heterogeneity) associated with the estimated pooled prevalence(s) reported in this study. However, this was minimized through estimating the pooled prevalence using a random-effects model and performing extensive subgroup and meta-regression analyses. Third, we acknowledge the limited number of studies from sub-Saharan Africa, and the pooled estimate from the few available studies might be overestimated. However, this provides an opportunity for further research on usage of herbal medicine among patients with cancer in Africa. Fourth, we only included studies published in the English language (thus missing studies published in other languages, particularly from the Francophone or Portuguese speaking countries) and did not include grey literature (dissertations or conference abstracts), which might have affected the outcomes (pooled prevalence rates) reported. Nonetheless, based on examination of the funnel plots and use of Egger's test of funnel plot asymmetry, no evidence of small-study effects (publication bias) was observed (found) across a sample of primary studies included in this study; therefore, the results of this review are unlikely to reflect bias. The majority of the included primary studies were prospective, and sufficient time was invested in these studies, making their results somewhat reliable. Finally, this study provides a strong point of reference for future studies, as it is one of the first reviews to be conducted on the prevalence of the use of herbal medicine amongst cancer patients.

## 5. Conclusion

This systematic review shows that a large percentage of patients with cancer use herbal medicine, especially those from low- and middle-income countries. In addition, larger percentages of adult patients with cancer (compared with children) and female patients with cancer (compared with males) used herbal medicine. In summary, there is need for additional epidemiological investigations exploring herbal medicine integration into cancer care especially for low- and middle-income countries.

## Figures and Tables

**Table 1:** Inclusion and exclusion criteria for included studies.

	Inclusion criteria	Exclusion criteria
Participants	Female and male participants of all ages suffering from cancer using herbal medicine(s) with or without any other CAM remedy(s) or conventional remedy(s).	Cancer survivors (recovered)
Outcomes	Prevalence of herbal medicine use (either reported or self-reported) Herbs commonly used by patients with cancer	Prevalence not reported
Study design	Cohort studies Cross-sectional studies	Expert reviews Case-control studies Policy reports Case studies Studies with aggregated CAM data
Ethical approval	Studies that were approved by an ethical review body or committee and participants consented to participate.	Lack of ethical approval and participant consent
Language	Published in the English language	Published in any other language

**Table 2:** Characteristics of included studies (*n* = 155).

Variable	Mean ± standard deviation or *n* (%)
Female (*n* = 96)	53.95 ± 14.04
Age, years (*n* = 86)	50.98 ± 17.39
Response rate (*n* = 99)	76.95 ± 19.78
Study year (10-year block)	
2000–2010	80 (51.61)
2010–2020	75 (48.39)
Region (continent)	
America	53 (34.19)
Asia	46 (29.68)
Europe	47 (30.32)
Oceania	6 (3.87)
Africa	3 (1.94)
Subregion (World Bank category)	
Europe and Central Asia	51 (32.92)
North America	47 (30.32)
East Asia and Pacific	36 (23.23)
Middle East	13 (8.39)
Latin America and the Caribbean	5 (3.23)
Sub-Saharan Africa	3 (1.94)
Income (World Bank category)	
Low/middle income	51 (34.23)
High income	98 (65.77)
Key individual countries (≥5 studies)	
USA	42 (27.10)
Turkey	18 (11.61)
Taiwan	7 (4.52)
UK	9(5.81)
China	7 (4.52)
Germany	5 (3.23)
Malaysia	5 (3.23)
Australia	5 (3.23)
Canada	5 (3.23)
Study design	
Cross-sectional/survey	154 (99.35)
Cohort	1 (0.65)
Study type/focus	
CAM	143 (92.26)
Herbal medicine only	12 (7.74)
Study setting	
Hospital (e.g., cancer clinic, institute, center, unit)	133 (85.81)
Cancer/tumor registry	6 (3.87)
General population (e.g., online)	16 (10.32)
Study population	
Adults	129 (83.23)
Children	23 (14.84)
Both	3 (1.94)
Sampling method	
Convenience	99 (63.87)
Consecutive	38 (24.52)
Simple random sampling	13 (8.39)
Quota	1 (0.65)
Systematic	1 (0.65)
Stratified	2 (1.29)
Multistage sampling	1 (0.65)
Data collection methods	
Self-report/self-administered interview	82 (52.90)
Researcher-administered interview (telephone, in-person)	59 (38.06)
Record/document review	6 (4.11)
Others (mixed, unclear)	8 (5.17)
Cancer type	
Breast	29 (18.71)
Prostate	13 (8.39)
Mixed (several types)	113 (72.90)

## Data Availability

The data used to support the findings of this study are included within the article and will be available online after publication of the article.

## Supplementary Materials

Supplementary Materials Supplementary data to this article are submitted together with the manuscript and are available online.
