# Supplementary material for: Prevalence of the Use of Herbal Medicines among Patients with Cancer: A Systematic Review and Meta-Analysis
Source: Evid Based Complement Alternat Med. 2021 May 17;2021:9963038. doi: 10.1155/2021/9963038 (PMC8149249; doi:10.1155/2021/9963038)
Supplement: Supplementary Materials — Supplementary data to this article are submitted together with the manuscript and are available online. [file 9963038.f1.docx]

**Supplementary Files**

Supplementary Table 1. PRISMA checklist for meta-analyses of studies

Supplementary Table 2. Search strategy for article selection (PubMed)

Supplementary Table 3. World Bank country and income group ranking of countries where the included studies were conducted.

Supplementary Table 4. Quality assessment criteria of selected articles

Supplementary Table 5. Quality assessment of selected articles

Supplementary Table 6. Other herbs used in cancer as reported by primary studies

Supplementary Table 1. PRISMA checklist for meta-analyses of studies

| **Section/topic** | **#** | **Checklist item** | **Reported on page #** |
| --- | --- | --- | --- |
| **TITLE** | | |  |
| Title | 1 | Identify the report as a systematic review, meta-analysis, or both. | **1** |
| **ABSTRACT** | | |  |
| Structured summary | 2 | Provide a structured summary including, as applicable: background; objectives; data sources; study eligibility criteria, participants, and interventions; study appraisal and synthesis methods; results; limitations; conclusions and implications of key findings; systematic review registration number. | **2** |
| **INTRODUCTION** | | |  |
| Rationale | 3 | Describe the rationale for the review in the context of what is already known. | **3-5** |
| Objectives | 4 | Provide an explicit statement of questions being addressed with reference to participants, interventions, comparisons, outcomes, and study design (PICOS). | **5** |
| **METHODS** | | |  |
| Protocol and registration | 5 | Indicate if a review protocol exists, if and where it can be accessed (e.g., web address), and, if available, provide registration information including registration number. | **6** |
| Eligibility criteria | 6 | Specify study characteristics (e.g., PICOS, length of follow-up) and report characteristics (e.g., years considered, language, publication status) used as criteria for eligibility, giving rationale. | **6** |
| Information sources | 7 | Describe all information sources (e.g., databases with dates of coverage, contact with study authors to identify additional studies) in the search and date last searched. | **7** |
| Search | 8 | Present full electronic search strategy for at least one database, including any limits used, such that it could be repeated. | **7** |
| Study selection | 9 | State the process for selecting studies (i.e., screening, eligibility, included in systematic review, and, if applicable, included in the meta-analysis). | **7** |
| Data collection process | 10 | Describe method of data extraction from reports (e.g., piloted forms, independently, in duplicate) and any processes for obtaining and confirming data from investigators. | **7** |
| Data items | 11 | List and define all variables for which data were sought (e.g., PICOS, funding sources) and any assumptions and simplifications made. | **7** |
| Risk of bias in individual studies | 12 | Describe methods used for assessing risk of bias of individual studies (including specification of whether this was done at the study or outcome level), and how this information is to be used in any data synthesis. | **8** |
| Summary measures | 13 | State the principal summary measures (e.g., risk ratio, difference in means). | **8** |
| Synthesis of results | 14 | Describe the methods of handling data and combining results of studies, if done, including measures of consistency (e.g., *I^2^*) for each meta-analysis. | **8** |

Page 1 of 2

| **Section/topic** | **#** | **Checklist item** | **Reported on page #** |
| --- | --- | --- | --- |
| Risk of bias across studies | 15 | Specify any assessment of risk of bias that may affect the cumulative evidence (e.g., publication bias, selective reporting within studies). | **N/A** |
| Additional analyses | 16 | Describe methods of additional analyses (e.g., sensitivity or subgroup analyses, meta-regression), if done, indicating which were pre-specified. | **8** |
| **RESULTS** | | |  |
| Study selection | 17 | Give numbers of studies screened, assessed for eligibility, and included in the review, with reasons for exclusions at each stage, ideally with a flow diagram. | 9-10 |
| Study characteristics | 18 | For each study, present characteristics for which data were extracted (e.g., study size, PICOS, follow-up period) and provide the citations. | 10-12 |
| Risk of bias within studies | 19 | Present data on risk of bias of each study and, if available, any outcome level assessment (see item 12). | Table 4 & 5 |
| Results of individual studies | 20 | For all outcomes considered (benefits or harms), present, for each study: (a) simple summary data for each intervention group (b) effect estimates and confidence intervals, ideally with a forest plot. | 12-24 |
| Synthesis of results | 21 | Present results of each meta-analysis done, including confidence intervals and measures of consistency. | 12-24 |
| Risk of bias across studies | 22 | Present results of any assessment of risk of bias across studies (see Item 15). | N/A |
| Additional analysis | 23 | Give results of additional analyses, if done (e.g., sensitivity or subgroup analyses, meta-regression [see Item 16]). | 12-24 |
| **DISCUSSION** | | |  |
| Summary of evidence | 24 | Summarize the main findings including the strength of evidence for each main outcome; consider their relevance to key groups (e.g., healthcare providers, users, and policy makers). | 25-29 |
| Limitations | 25 | Discuss limitations at study and outcome level (e.g., risk of bias), and at review-level (e.g., incomplete retrieval of identified research, reporting bias). | 29-30 |
| Conclusions | 26 | Provide a general interpretation of the results in the context of other evidence, and implications for future research. | 31 |
| **FUNDING** | | |  |
| Funding | 27 | Describe sources of funding for the systematic review and other support (e.g., supply of data); role of funders for the systematic review. | 31 |

***Note****. From:* Moher D, Liberati A, Tetzlaff J, Altman DG, the PRISMA Group. (2009). Preferred Reporting Items for Systematic Reviews and Meta-Analyses: The PRISMA Statement. PLoS Med 6(7): e1000097. doi:10.1371/journal.pmed1000097. (**www.prisma-statement.org)**.

Supplementary Table 2. Search strategy for article selection (PUBMED)

| **Search** | **Query** |
| --- | --- |
| #4 | Search ((((((((Cancer) OR Malignancy) OR Tumo*) OR Neoplasm) AND (“2000/01/01”[PDat]: “2020/01/01”[PDat]))) AND (((((herbs) OR herbal medicine) OR herbal material) OR herbal preparation) AND (“2000/01/01”[PDat]: “2020/01/01”[PDat]))) AND (“2000/01/01”[PDat]: “2020/01/01”[PDat]))) AND (((((observational) OR cross-sectional) OR survey) OR cohort) AND (“2000/01/01”[PDat]: “2020/01/01”[PDat])) Filters: Publication date from 2000/01/01 to 2020/01/01 Sort by: [pubsolr12] |
| #3 | Search (((observational) OR cross-sectional) OR survey) OR cohort Filters: Publication date from 2000/01/01 to 2020/01/01 Sort by: [pubsolr12] |
| #2 | Search (((herbs) OR herbal medicine) OR herbal material) OR herbal preparation Filters: Publication date from 2000/01/01 to 2020/01/01 Sort by: [pubsolr12] |
| #1 | Search (((Cancer) OR Malignancy) OR Tumo*) OR Neoplasm Filters: Publication date from 2000/01/01 to 2020/01/01 Sort by: [pubsolr12] |

Supplementary Table 3. World Bank country and income group ranking of countries where the included studies were conducted ([www.worldbank.org](http://www.worldbank.org))

| **Country** | **Continent** | **Sub-region** | **Income group** |
| --- | --- | --- | --- |
| 1. UK | Europe | Europe and Central Asia | High income |
| 1. China | Asia | East Asia and Pacific | Upper middle income |
| 1. Ghana | Africa | Sub-Saharan Africa | Lower middle income |
| 1. Turkey | Europe | Europe and Central Asia | Upper middle income |
| 1. USA | America | North America | High income |
| 1. Thailand | Asia | East Asia and Pacific | Upper middle income |
| 1. Mongolia | Asia | East Asia and Pacific | Lower middle income |
| 1. Ethiopia | Africa | Sub-Saharan Africa | Low income |
| 1. Japan | Asia | East Asia and Pacific | High income |
| 1. Taiwan | Asia | East Asia and Pacific | High income |
| 1. Germany | Europe | Europe and Central Asia | High income |
| 1. Switzerland | Europe | Europe and Central Asia | High income |
| 1. Saudi Arabia | Asia | Middle East | High income |
| 1. Trinidad and Tobago | America | Latin America and the Caribbean | High income |
| 1. Mexico | America | Latin America and the Caribbean | Upper middle income |
| 1. South Korea | Asia | East Asia and Pacific | High income |
| 1. Norway | Europe | Europe and Central Asia | High income |
| 1. Croatia | Europe | Europe and Central Asia | High income |
| 1. Singapore | Asia | Europe and Central Asia | High income |
| 1. Italy | Europe | Europe and Central Asia | High income |
| 1. Malaysia | Asia | Europe and Central Asia | Upper middle income |
| 1. Nigeria | Africa | Sub-Saharan Africa | Lower middle income |
| 1. Lebanon | Asia | Middle East | Upper middle income |
| 1. New Zealand | Oceania | East Asia and Pacific | High income |
| 1. Canada | America | North America | High income |
| 1. Uruguay | America | Latin America and the Caribbean | High income |
| 1. Iran | Asia | Middle East | Upper middle income |
| 1. Australia | Oceania | East Asia and Pacific | High income |
| 1. Estonia | Europe | Europe and Central Asia | High income |
| 1. Guatemala | America | Latin America and the Caribbean | Upper middle income |
| 1. Israeli | Europe | Middle East | High income |
| 1. Denmark | Europe | Europe and Central Asia | High income |
| 1. Hungary | Europe | Europe and Central Asia | High income |
| 1. Netherland | Europe | Europe and Central Asia | High income |
| 1. Palestine | Asia | Middle East | Lower middle income |
| 1. Jordan | Asia | Middle East | Upper middle income |
| 1. Argentina | America | Latin America and the Caribbean | Upper middle income |
| 1. Greece | Europe | Europe and Central Asia | High income |
| 1. Sweden | Europe | Europe and Central Asia | High income |
| 1. Serbia | Europe | Europe and Central Asia | Upper middle income |
| 1. Czech Republic | Europe | Europe and Central Asia | High income |
| 1. Belgium | Europe | Europe and Central Asia | High income |
| 1. Iceland | Europe | Europe and Central Asia | High income |
| 1. Spain | Europe | Europe and Central Asia | High income |
| Specific countries under blocks but reported in separate studies | | | |
| UK |  |  |  |
| 1. Ireland | Europe | Europe and Central Asia | High income |
| 1. Scotland | Europe | Europe and Central Asia | High income |
| US |  |  |  |
| 1. Hawaii | America | North America | High income |

***Note***: Low-income economies classified as gross national income (GNI) per capita of $1,025 or less in 2018, lower middle-income economies as a GNI per capita between $1,026 and $3,995; upper middle-income economies as a GNI per capita between $3,996 and $12,375; high-income economies as a GNI per capita of $12,376 or more ([www.worldbank.org](http://www.worldbank.org)).

Supplementary Table 4. Quality assessment criteria of selected articles

| **Bias type** | **Low risk of bias** | **Moderate risk of bias (unclear) risk of bias** | **High risk of bias** |
| --- | --- | --- | --- |
| Selection (sample population) | Participants selected randomly/used probability sampling method or used any other non-probability sampling method, but endeavored to include all possible participants. | Insufficient information or not reported | Used non-probability sampling method |
| Selection (participation rate) | High participation rate (70%–85%) | Insufficient information or not reported | Low participation rate (<70%) |
| Performance bias (analytical methods to control for bias) | Adjusted for cofounding | Did not clearly indicate if they adjusted for cofounding | Did not adjust for confounding |

Supplementary Table 5. Quality assessment of selected articles

|  |  | **Selection Bias** |  | **Performance Bias** |
| --- | --- | --- | --- | --- |
| **Study ID** | **Author** | **Sample population** | **Participation/response rate bias** | **Performance/confounding bias** |
| 1 | Damery (2011) | High risk | Low risk | Low risk* |
| 2 | Liu (2012) | High risk | High risk | Low risk* |
| 3 | Yarney (2013) | High risk | Low risk | Low risk |
| 4 | Yalcin (2017) | High risk | Moderate risk | Low risk |
| 5 | Tas (2005) | High risk | Moderate risk | Low risk |
| 6 | Osian (2015) | High risk | Moderate risk | High risk |
| 7 | Peltzer (2019) | High risk | Low risk | Low risk |
| 8 | Oyunchimeg (2017) | High risk | Low risk | Low risk |
| 9 | Kucukoner (2012) | High risk | Moderate risk | High risk |
| 10 | Gulluoglu (2008) | Low risk | Moderate risk | Low risk |
| 11 | Erku (2016) | High risk | Low risk | Low risk |
| 12 | Chen (2008) | Low risk | Low risk | Low risk |
| 13 | Lee (2000) | High risk | Low risk | Low risk |
| 14 | Hyodo (2005) | Moderate risk | High risk | Low risk |
| 15 | Molassiotis (2006) | High risk | Moderate risk | High risk |
| 16 | Kuo (2018) | Low risk | Low risk | Low risk |
| 17 | Lin (2011) | High risk | Low risk | High risk |
| 18 | Molassiotis (2005) | High risk | Moderate risk | Low risk |
| 19 | Tautz (2012) | High risk | Low risk | High risk |
| 20 | Jermini (2019) | High risk | Moderate risk | High risk |
| 21 | Cilingir (2017) | High risk | Low risk | High risk |
| 22 | Kao (2000) | High risk | Low risk | High risk |
| 23 | Rabia (2009) | High risk | Moderate risk | High risk |
| 24 | Abuelgasim (2018) | High risk | Moderate risk | Low risk |
| 25 | Bahall (2017) | High risk | Low risk | Low risk |
| 26 | Corner (2009) | Low risk | High risk | High risk |
| 27 | Gomez-Martinez (2007) | High risk | Moderate risk | High risk |
| 28 | Inanç (2006) | High risk | Moderate risk | High risk |
| 29 | Jang (2017) | Low risk | Low risk | Low risk |
| 30 | Kristoffersen (2019) | High risk | High risk | Low risk |
| 31 | Kust (2016) | High risk | Moderate risk | Low risk |
| 32 | Molassiotis (2006) | High risk | Moderate risk | High risk |
| 33 | Tarhan (2009) | High risk | Moderate risk | High risk |
| 34 | Wong (2010) | High risk | Low risk | High risk |
| 35 | Yildiz (2013) | High risk | High risk | Low risk |
| 36 | Berretta (2017) | High risk | Moderate risk | High risk |
| 37 | Bonacchi (2014) | Low risk | Low risk | Low risk |
| 38 | Dhanoa (2014) | High risk | Low risk | Low risk |
| 39 | Ezeome (2007) | Low risk | Low risk | High risk |
| 40 | Greenlee (2009) | High risk | Moderate risk | Low risk* |
| 41 | Zulkipli (2018) | High risk | Low risk | Low risk |
| 42 | Shih (2009) | High risk | Moderate risk | High risk |
| 43 | Puataweepong (2011) | Low risk | Low risk | High risk |
| 44 | Judson (2017) | High risk | High risk | High risk |
| 45 | Link (2013) | High risk | High risk | Low risk* |
| 46 | Magi (2015) | Low risk | High risk | High risk |
| 47 | Mceachrane-Gross (2006) | Low risk | High risk | High risk |
| 48 | Naja (2017) | High risk | Low risk | Low risk |
| 49 | Nazik (2012) | High risk | Moderate risk | High risk |
| 50 | Chrystal (2003) | High risk | High risk | High risk |
| 51 | Naja (2015) | High risk | Low risk | Low risk |
| 52 | Pourtsidis (2015) | High risk | High risk | High risk |
| 53 | Supoken (2009) | High risk | Moderate risk | High risk |
| 54 | Shen (2002) | High risk | Moderate risk | Low risk |
| 55 | Yap (2004) | High risk | Low risk | High risk |
| 56 | Rocha (2017) | Low risk | Moderate risk | Low risk |
| 57 | Gross (2007) | High risk | Low risk | Low risk |
| 58 | Paisley (2011) | Low risk | Low risk | High risk |
| 59 | Farooqui (2016) | High risk | Moderate risk | High risk |
| 60 | Karadeniz (2007) | High risk | Moderate risk | High risk |
| 61 | Koçaşlı (2017) | High risk | Moderate risk | High risk |
| 62 | Kuo (2018) | Low risk | Low risk | Low risk |
| 63 | Lam (2009) | Low risk | Low risk | High risk |
| 64 | Martel (2005) | High risk | Low risk | High risk |
| 65 | Montazeri (2006) | Low risk | Moderate risk | Low risk |
| 66 | Sanchez (2015) | High risk | Moderate risk | High risk |
| 67 | Salminen (2004) | High risk | Moderate risk | Low risk |
| 68 | Singh (2005) | High risk | Moderate risk | High risk |
| 69 | Hwang (2015) | Low risk | Low risk | Low risk |
| 70 | Werneke (2004) | High risk | High risk | High risk |
| 71 | Huebner (2014) | High risk | Low risk | High risk |
| 72 | Teng (2010) | High risk | Low risk | High risk |
| 73 | Mcquade (2012) | High risk | Low risk | High risk |
| 74 | Maskarinec (2000) | High risk | High risk | Low risk |
| 75 | Al-Naggar (2013) | High risk | Moderate risk | High risk |
| 76 | Pihlak (2013) | High risk | Moderate risk | High risk |
| 77 | Catt (2006) | High risk | Moderate risk | High risk |
| 78 | Kessel (2016) | High risk | High risk | High risk |
| 79 | Ladas (2014) | High risk | Moderate risk | Low risk |
| 80 | Lin (2010) | High risk | Low risk | High risk |
| 81 | Gozum (2007) | High risk | Moderate risk | High risk |
| 82 | Yoshimura (2003) | High risk | Low risk | High risk |
| 83 | Arush (2006) | Low risk | Low risk | High risk |
| 84 | O’connor (2013) | Low risk | High risk | High risk |
| 85 | Wells (2007) | High risk | Low risk | Low risk |
| 86 | Powell (2002) | High risk | Low risk | High risk |
| 87 | Vapiwala (2006) | High risk | Low risk | High risk |
| 88 | Wyatt (2010) | Low risk | Low risk | Low risk |
| 89 | Cui (2004) | High risk | Low risk | High risk |
| 90 | Hunter (2014) | High risk | Low risk | High risk |
| 91 | Rees (2000) | Low risk | Low risk | Low risk |
| 92 | Lynda (2006) | Low risk | Moderate risk | High risk |
| 93 | Albabtain (2018) | High risk | High risk | Low risk |
| 94 | Algier (2005) | High risk | Moderate risk | High risk |
| 95 | Ashikaga (2002) | High risk | High risk | Low risk |
| 96 | Chan (2005) | High risk | Moderate risk | Low risk |
| 97 | Chao (2014) | High risk | Low risk | High risk |
| 98 | Ferro (2007) | High risk | Moderate risk | Low risk |
| 99 | Kang (2012) | High risk | Low risk | Low risk |
| 100 | Klafke (2011) | High risk | Low risk | Low risk |
| 101 | Loquai (2017) | High risk | Low risk | Low risk |
| 102 | Molassiotis (2006) | High risk | Moderate risk | High risk |
| 103 | Pedersen (2009) | High risk | High risk | Low risk |
| 104 | Sárváry (2019) | High risk | Low risk | High risk |
| 105 | Scott (2005) | High risk | Moderate risk | High risk |
| 106 | Singendonk (2013) | Low risk | Low risk | Low risk |
| 107 | Tomlinson (2011) | High risk | High risk | Low risk |
| 108 | Tsai (2000) | High risk | Low risk | High risk |
| 109 | Patterson (2002) | Low risk | Low risk | Low risk |
| 110 | Yildirim (2010) | High risk | Low risk | High risk |
| 111 | Jones (2002) | High risk | Low risk | High risk |
| 112 | Mclay (2012) | High risk | Low risk | High risk |
| 113 | Molassiotis (2005) | High risk | Moderate risk | Low risk |
| 114 | Molassiotis (2005) | High risk | Moderate risk | High risk |
| 115 | Neuhouser (2001) | Moderate risk | High risk | High risk |
| 116 | Engdal (2008) | High risk | Low risk | Low risk |
| 117 | Chen (2015) | High risk | Low risk | High risk |
| 118 | Can (2009) | High risk | Low risk | Low risk |
| 119 | Ali-Shtayeh (2011) | High risk | Moderate risk | High risk |
| 120 | Johannessen (2008) | High risk | Low risk | High risk |
| 121 | Pud (2005) | High risk | Moderate risk | High risk |
| 122 | Sadatbazrafshani (2019) | High risk | Moderate risk | Low risk* |
| 123 | Jaradat (2016) | High risk | Low risk | Low risk |
| 124 | Richardson (2000) | High risk | High risk | Low risk |
| 125 | Afifi (2010) | Low risk | Moderate risk | High risk |
| 126 | Lin (2011) | Low risk | Low risk | Low risk |
| 127 | Chui (2014) | Low risk | Low risk | Low risk |
| 128 | Lin (2012) | Low risk | Low risk | High risk |
| 129 | Byeongsang Oh (2010) | Low risk | High risk | High risk |
| 130 | Bauml (2014) | High risk | Low risk | Low risk |
| 131 | Arthur (2013) | Low risk | Low risk | High risk |
| 132 | Greenlee (2016) | High risk | High risk | Low risk |
| 133 | Ali-Shtayeh (2016) | Low risk | Moderate risk | High risk |
| 134 | Ali-Shtayeh (2016) | Low risk | Moderate risk | High risk |
| 135 | Luo (2016) | High risk | High risk | Low risk |
| 136 | Eng (2003) | High risk | High risk | High risk |
| 137 | Hann (2006) | Low risk | Moderate risk | Low risk |
| 138 | Davis (2006) | High risk | High risk | Low risk |
| 139 | Guethlin (2010) | High risk | Low risk | High risk |
| 140 | Rosen (2013) | High risk | High risk | Low risk |
| 141 | Molassiotis (2004) | High risk | High risk | High risk |
| 142 | Kelly (2000) | High risk | Low risk | High risk |
| 143 | Mccurdy (2003) | High risk | Low risk | Low risk |
| 144 | Ball (2005) | High risk | Moderate risk | Low risk |
| 145 | Hall (2003) | High risk | Moderate risk | High risk |
| 146 | Wiygul (2005) | High risk | High risk | High risk |
| 147 | Hann (2006) | High risk | High risk | High risk |
| 148 | Ilknur Aydin (2009) | High risk | Low risk | High risk |
| 149 | Zeliha Koc (2011) | High risk | Low risk | High risk |
| 150 | Leng (2014) | High risk | High risk | High risk |
| 151 | Kakai (2003) | Low risk | Low risk | High risk |
| 152 | Bismarck (2014) | High risk | High risk | Low risk |
| 153 | Smith (2016) | High risk | Low risk | Low risk |
| 154 | Anderson (2012) | Low risk | High risk | Low risk |

***Note.*** Selection bias: specific ethical issues involved in obtaining a list of patients with cancer (sampling frame) may have made random sampling impossible, therefore most authors opted for consecutive/convenient/purposive sampling. In addition, the majority of the studies with low response rates had large sample sizes (>100 participants). As the majority of the studies were about complementary and alternative therapies use, control for confounding during multivariate analysis was as well conducted, consistent with use of complementary and alternative therapies and not specifically with herbal medicine use, making assessment of this parameter partially relevant. Lastly, as the majority of the studies measured the outcome (herbal medicine use) through self-report (same method), quality assessment by how an outcome was measured was considered for this study.

Supplementary Table 6. Other herbs used in cancer as reported in the primary studies

| **Other herbs used in cancer as reported by participants in primary studies** |
| --- |
| Radish (Raphanus sativus), Chasteberry (Vitex agnus castus), Dill (Anethum graveolens), Arnica (Arnica montana), Starflower (Borago officinalis), Daisy (Bellis perennis), Juniper (Juniperus), Avens (Geum urbanum), Dong quai (Angelica sinensis), Bistort (Polygonum bistorta), Pewter grass (Equisetum arvense), Almond (Prunus amygdalus), Red vine leaf (Vitis vinifera), Plantain (Plantago), Mallow (Malva sylvestris), Germander (Teucrium chamaedrys), Carob (Ceratonia siliqua), Celery (Apium graveolens), Centaury (Centaurea acaulis), Cinchona bark (Cinchona) Parsley (Carum petroselinum), Willow (Salix alba), Yarrow (Achillea millefolium), Common fennel (Foeniculum vulgare), Black cohosh, Coltsfoot (Tussilago farfara), Couch grass (Agropyron repens), Calendula (Calendula officinalis), Habbatus sauda seeds, Cascara sagrada, Broccoli (Brassica oleracea italica), Dead-nettle (Lamium album), Burdock, Cat’s claw (Uncaria tomentosa), Harmal seeds (Peganum harmala), Dandelion, Lemon balm (Melissa officinalis), Feverfew (Tanacetum parthenium), Hawthorne, Bach flower remedies, Lime flower (Tiliae flos), Long pepper (Piper longum), Hibiscus (Hibiscus rosa-sinensis), Miltwaste (Asplenium Ceterach), Mountain ash (Sorbus aucuparia), Cranberry, Rose mallow (Alcea rosea), Blue cohosh, Black briar, Saffron (Crocus sativus), Bromelain, Horse chestnut, Chicory (Cichorium intybus), Carrot juice (Daucus carota), Lathyrus, Sweet basil (Ocimum basilicum), Orange blossom (Citrus aurantium), Camphor (Cinnamomum camphora), Apricot seeds, Atractylodes, Caper (Capparis spinosa), Astragalus root, Hedyotis diffusa, Bilberry, Scutellaria, Passion flower, Barbata (Ban Zhi Lian), Borage seed oil, Ephedra, Anise, Blueberry extract, Cactus, Carqueja, Graviola, Bird’s-foot (Lotus corniculatus), Arnica, Valerian, Slippery elm, Caulis spatholobi (Jixueteng), Salvia miltiorrhiza radix (Dan Shen) and Radix astragali (Huangqi), Oldenlandia (Bai Hua She She Cao), Beetroot, Sea buckthorn, Balsam cucumber (Momordica charantia), Equisetum arvense, Mangosteen, Ashwagandha, Linseed, Bee pollen, Cirsium arvense, Artichoke extract, Berry extract, Black walnut, and Red clover. |
